# Magnetic interactions between metal nanostructures within porous silicon

**DOI:** 10.1186/1556-276X-9-412

**Published:** 2014-08-21

**Authors:** Klemens Rumpf, Petra Granitzer, Nobuyoshi Koshida, Peter Poelt, Michael Reissner

**Affiliations:** 1 Institute of Physics, Karl Franzens University Graz, Universitaetsplatz 5, Graz 8010, Austria; 2 Graduate School of Engineering, Tokyo University of Agriculture and Technology, Tokyo 184-8588, Japan; 3 Institute for Electron Microscopy, University of Technology Graz, Graz 8010, Austria; 4 Institute of Solid State Physics, Vienna University of Technology, Wiedner Hauptstr. 8, Vienna 1040, Austria

**Keywords:** Porous silicon, Electrodeposition, Magnetic nanostructures, Magnetic interactions

## Abstract

Electrochemically deposited magnetic nanostructures arranged in a three-dimensional system are investigated with respect to their cross-talk between each other. The nanostructures are embedded in porous silicon templates with different morphologies which means pores offering dendritic growth of different strengths. An increase of the uniformity of the pores is concomitant with an increase of the smoothness of the metal deposits which strongly influences the magnetic behavior of the system. Less dendritic structures lead to an increase of the coercivity of the nanocomposite which reveals less cross-talk between the metal deposits due to a modification of the stray fields. The system allows in a cheap and simple way to tune the magnetic interactions of magnetic nanostructures in a three-dimensional arrangement.

**PACS:**

81.05.Rm; 81.07.Gf; 75.75.-c

## Background

The adjustability of magnetic properties of nanostructured magnets and magnetic nanocomposite systems is a crucial point in today's research. In general, the magnetic properties of such systems depend on the used magnetic material, the shape of the nanostructures, and also on their mutual arrangement. Three-dimensional arrays of magnetic nanostructures are often a favorable composition also in terms of miniaturization. In three-dimensional systems, magnetic dipolar coupling between neighboring nanostructures has to be considered dependent on the distance between each other.

Porous silicon is tunable in its morphology, and it is therefore a versatile host material for the incorporation of various materials into the pores. Not only the infiltration of molecules [1] or nanoparticles [2] but also the deposition of different metals [3] within the pores can be carried out. The deposition of magnetic materials results in a semiconducting/ferromagnetic nanocomposite with tunable magnetic properties. In the following, the deposition of Ni-wires within self-organized porous silicon offering different morphologies will be elucidated, and the magnetic properties in dependence on the morphology of the pores (and embedded Ni-wires) will be discussed.

## Methods

### Experimental results

Porous silicon templates with different pore diameters and with different dendritic pore growths have been created by anodization of n^+^-silicon in aqueous hydrofluoric acid solution. The morphology of porous silicon can be controlled in a broad range by the electrochemical conditions. In this case, different morphologies are fabricated by varying the current density applied for the anodization process. Details about this pore-formation process can be found elsewhere [4]. The pore-diameters have been decreased from an average value of 90 to 30 nm which results in an increase of the side-pore length from about 20 nm to about 50 nm. The concomitant mean distance between the pores increases with the decrease of the pore diameter from 40 to 80 nm, whereas the porosity of the porous layer decreases from about 80% to about 45%.

**Figure 1:**
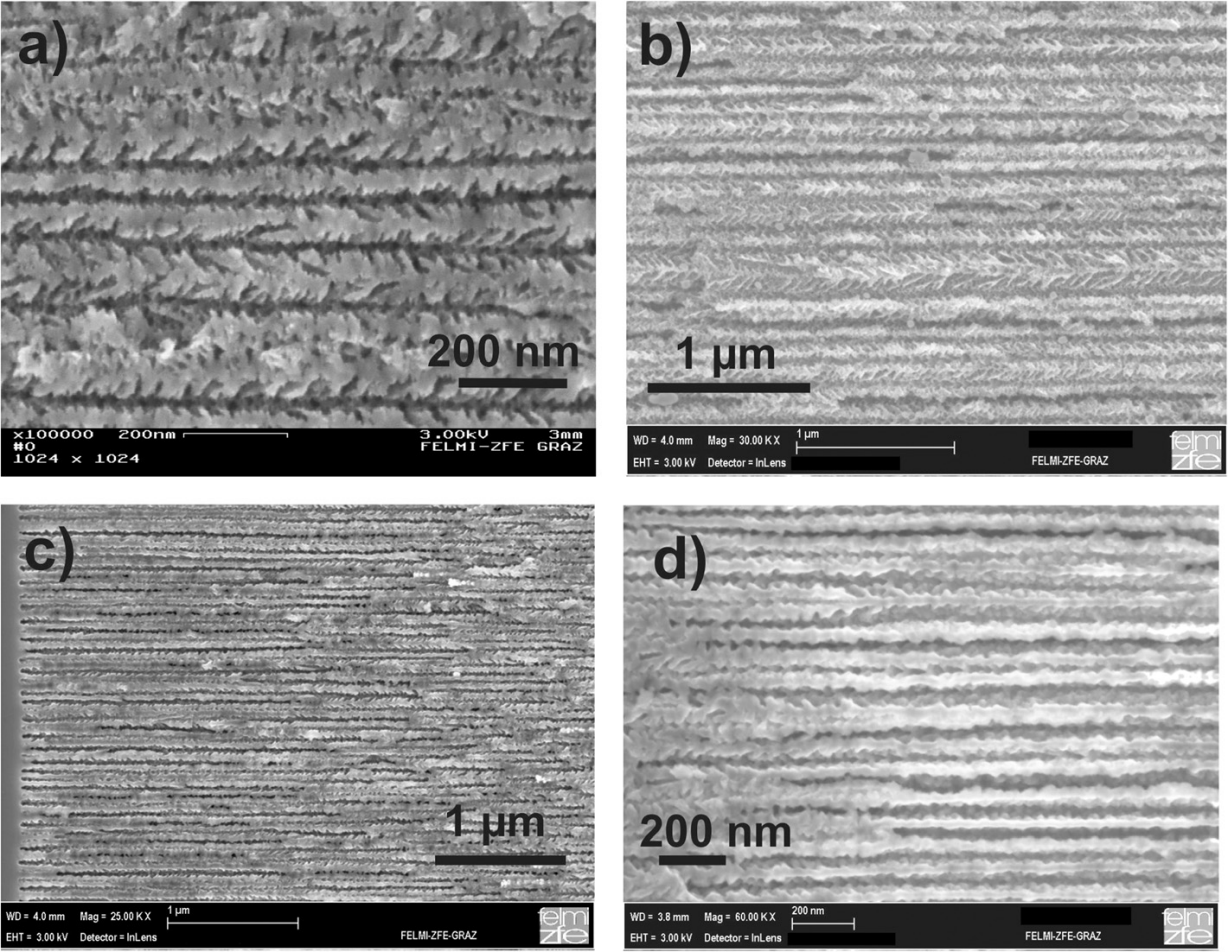
**Porous silicon templates fabricated by anodization offering different pore diameters.** A decrease of the dendritic pore growth with increasing pore diameter can be seen. **(a)** Average pore diameter 25 nm, **(b)** average pore diameter 80 nm. Samples **(c)** with a pore diameter of approximately 25 nm and **(d)** with a pore diameter of approximately 40 nm have been prepared by anodization during the application of a magnetic field of 8 T. The side pores are diminished significantly.

In employing a sophisticated method by applying an external magnetic field of 8 T perpendicular to the sample surface during the anodization process, an average pore diameter of 35 nm with very low dendritic growth (side-pore length below 10 nm) could be achieved [5]. Figure 1 shows three typical templates with a pore-diameter of 90 nm (side-pore length approximately 20 nm), 40 nm (side-pore length approximately 50 nm), and 35 nm (side-pore length <10 nm), whereas the latter sample has been prepared by magnetic field-assisted etching.

These porous silicon templates fabricated by the two different anodization processes have been filled with Ni-wires by electrodeposition. The filling factor of the samples ranges between 40 and 50%. The shape of the deposited Ni-wires corresponds to the shape of the pores and thus also exhibits an according branched structure.

Magnetization measurements have been carried out with a vibrating sample magnetometer (VSM, Quantum Design, San Diego, CA, USA) in the field range ±1 T and at a temperature of 300 K. The magnetic field has been applied parallel to the pores, which means easy axis magnetization.

## Results and discussion

**Figure 2:**
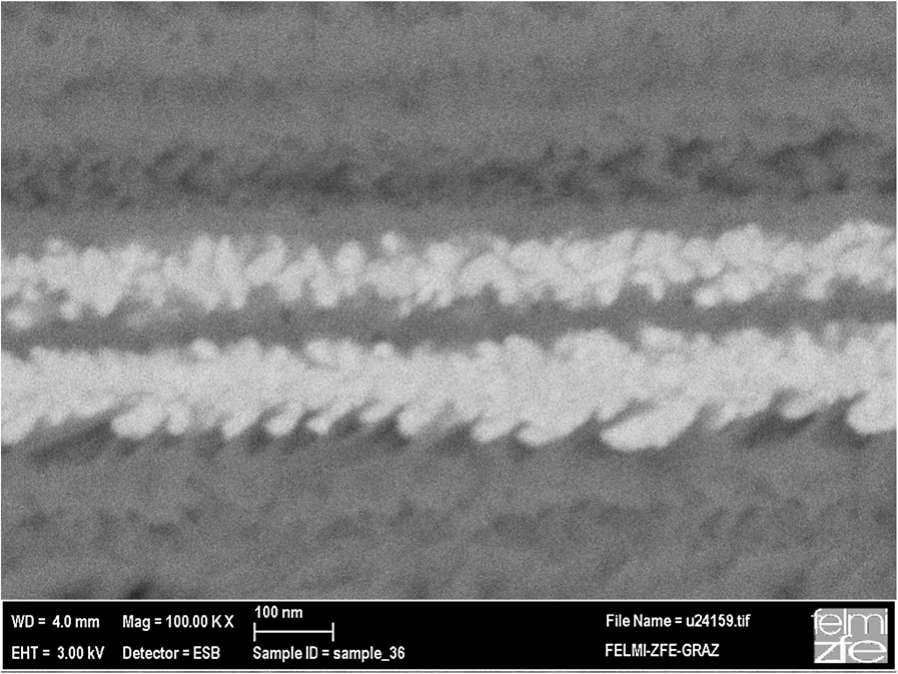
Backscattered electron (BSE) image showing deposited Ni-wires matching the morphology of the porous silicon structure.

The magnetic properties of Ni-nanowires embedded within the pores of porous silicon with different morphologies (different dendritic growths) are discussed in terms of dipolar coupling between adjacent wires. In the case of conventional etching, the pore diameter (wire-diameter) is varied between 90 and 30 nm, and the length of the wires is in all cases between 1 and 2 μm. Concomitant to the change of the pore diameter, the length of the side pores is modified between 20 and 50 nm. With decreasing pore diameter, the length of the side pores is increased. Nevertheless, in all investigated samples, the pores are clearly separated from each other. Figure 2 shows a porous silicon sample with an average pore diameter of 90 nm filled with Ni-wires. It can be seen that the deposited Ni matches the morphology of the pores.

In general, magnetic interactions between neighboring metal wires influence strongly the coercive fields and the remanence. Dipolar coupling between nanowires can reduce the coercivity of nanowire array significantly [6]. Also, the behavior of the magnetic moments within the wires is affected by the stray fields of the wires which perturb the magnetization reversal process of the wires [7].

**Figure 3:**
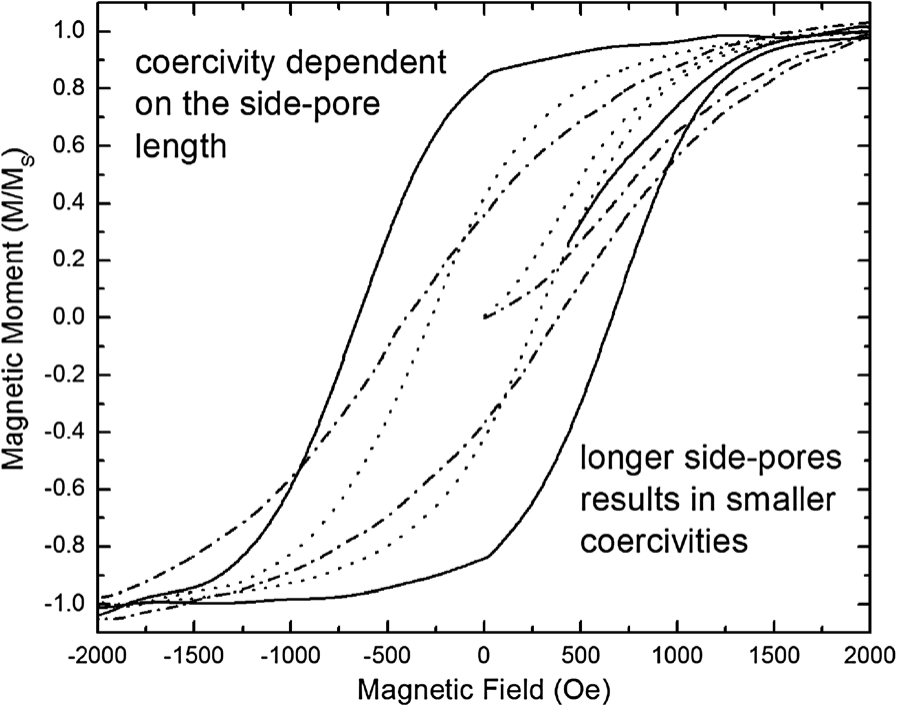
**Magnetization curves of porous silicon samples loaded with Ni-wires in terms of different dendritic growths.** The coercivity increases with decreasing side-pore length (dotted curve approximately 50 nm; dashed curve approximately 20 nm; full curve approximately 10 nm).

**Figure 4:**
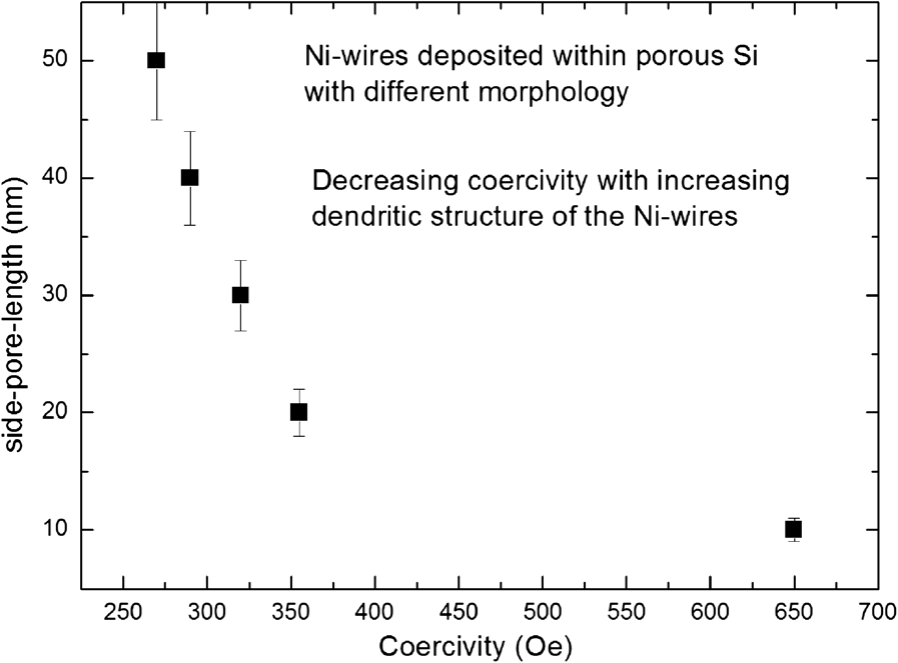
**Coercivity of Ni-filled porous silicon versus side-pore length of the templates.** Decreasing side-pore length is concomitant with an increase of the pore diameter (conventional etched samples). The sample offering a side-pore length of 10 nm has been prepared by magnetic field-assisted etching.

A decrease of the coercivity of a Ni-nanowire array has been observed by investigating samples with different porous morphologies. This decrease can be assigned to increasing magnetic interactions between neighboring wires caused by increasing side-pore length. Magnetic field-dependent measurements on the porous silicon/Ni composites which have been prepared by conventional etching show a decrease of the coercivity with decreasing pore diameter which can be varied between *H*_C_ = 450 Oe to *H*_C_ = 100 Oe, whereas the coercivity of the specimen prepared by magnetic field-assisted anodization offers a coercivity of *H*_C_ = 650 Oe which is much higher. Also, the magnetic remanence *M*_R_ decreases with increasing dendritic structure of the deposited Ni-wires. Magnetic field-assisted etched samples offer a remanence at least twice the value as in the case of conventional etched samples which results in a difference of the squareness (*M*_R_/*M*_S_) between 85 and 42%. In Figure 3, magnetic field-dependent measurements are presented showing the decrease of the coercivity with increasing roughness of the deposited Ni-wires. These results indicate that the magnetic coupling between neighboring Ni-wires decreases with decreasing dendritic pore growth because the effective distance between the pores is increased due to shorter side pores and also due to less contribution of the dendrites to the stray fields. Figure 4 shows the dependence of the coercivity on the side-pore length. In the case of conventional etched porous silicon with decreasing side-pore length from about 50 nm (pore diameter approximately 40 nm) to about 30 nm (pore diameter approximately 80 nm) and further to about 20 nm (pore diameter approximately 90 nm), an increase in the coercivity has been observed from *H*_C_ = 270 Oe to *H*_C_ = 320 Oe and to *H*_C_ = 355 Oe. Similar results of such low coercive field values of Ni-nanowire arrays have also been observed by other groups [8,9]. In the case of magnetic field-assisted etched porous silicon, an average pore diameter of 35 nm has been achieved, whereas the mean side-pore length is around 10 nm. The observed coercivity of such a sample is 650 Oe. The difference of the coercivity between Ni-wires deposited within conventional etched and magnetic field-assisted etched samples ranges between 45 and 58%. Simulations of arrays of nanowires show that dipolar coupling has to be taken into account if the distance between the wires is in the range of the wire diameter [10]. In the case of closely packed wires, the infinite wire approach has to be considered because the magnetization reversal of the wires is modified by the packing density [10].Figure 4 shows the coercivity in dependence on the length of the side pores of the porous silicon template and the length of the branches of the Ni-wires, respectively.

## Conclusions

A system consisting of a porous silicon host with different dendritic growths and embedded Ni-wires which offer a shape correlated to the pores has been presented. This nanocomposite offering a three-dimensional arrangement of Ni-nanowires has been produced in a cheap and simple way without any pre-structuring methods. The magnetic properties can also be tuned beside the employed metal and the shape of the deposits by the morphology of the host material. A decrease of the branched structure of the pores results in an increase of the coercivity which is due to less magnetic cross-talk between neighboring Ni-wires.

## Competing interests

The authors declare that they have no competing interests.

## Authors' contributions

KR and PG fabricated the samples by conventional etching and performed all the electrodeposition and also carried out the magnetization measurements. NK provided the magnetic field-assisted porous silicon samples. PP performed the SEM investigations. All authors discussed the data and prepared the manuscript. All authors read and approved the final manuscript.
